# Mitochondrial Microproteins: Emerging Regulators in Neurodevelopment and Neurodegeneration

**DOI:** 10.1002/bies.70058

**Published:** 2025-08-22

**Authors:** Nada Borghol, Sozerko Yandiev, Julien Courchet

**Affiliations:** ^1^ Univ Lyon Univ Lyon 1 CNRS INSERM Physiopathologie Et Génétique du Neurone et Du Muscle UMR5261 U1315 Institut NeuroMyoGène Lyon France; ^2^ Laboratory of Molecular Biology and Cancer Immunology Faculty of Science I Lebanese University Rafik Hariri Campus Hadat Lebanon

**Keywords:** metabolism, microproteins, mitochondria, neurodegeneration, neurodevelopment, neuron

## Abstract

Recent advances in genomics uncovered a large number of microproteins, which are peptides of less than 100 amino‐acids encoded by small open reading frames. In contrast to their identification, the validation of the functions of microproteins remains challenging. Especially, what are their biological functions in the cell and how this relates to disease conditions are still largely unknown. Although microproteins ensure a plethora of cellular functions, recent evidence demonstrate that they may disproportionately affect cellular metabolism. In this review, we will address the roles of mitochondrial‐targeted microproteins, and especially how this class of protein regulates neuronal metabolism in neurodevelopment and neurodegeneration, and may contribute to axonal and dendritic metabolic disorders.

## Graphical abstract

Mitochondrial microproteins have emerged as critical regulators of mitochondrial function. Neurons are highly dependent on mitochondria to sustain their function. Mitochondrial microproteins can be deregulated and can contribute to the pathogenesis of neurodegenerative disorders. Mitochondrial microproteins are involved in neuronal development. However, a more thorough experimental characterization is required. Figure created in BioRender (2025) https://BioRender.com.

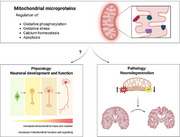

## 1 Introduction

With the advent of genome sequencing [1], genomic analyses first estimated the number of protein‐coding genes to 20.000 in the human genome. Advances in large‐scale transcriptomics uncovered a substantial number of transcripts originally qualified as “noncoding RNA” [2]. In the past two decades and with the development of innovative proteomic approaches, small Open Reading Frame (sORF)‐Encoded Peptides, also known as SEPs or microproteins, were identified and, in part, derived from the previously considered “noncoding RNA”, thereby adding another layer of complexity to the human proteome.

Historically, the identification of these small proteins was very limited by the sharp 300‐bases (100 codons) transcripts cut‐off implemented in ORF‐prediction algorithms [3]. Nevertheless, researchers provided evidence of thousands of putative microproteins during the last two decades, initially by using computational analysis and later by experimental validation via RIBO‐seq, Mass Spectrometry (MS), and proteogenomics approaches where MS is coupled to OMICS data for further validation. Now, sORF data are compiled into publicly available databases, allowing their identification [4]. Efforts are being deployed in deciphering the functional features of sORF‐derived microproteins, but only a limited number of these have had their functions revealed. They exert a wide variety of roles in biological processes and diseases, both in prokaryotes and eukaryotes [5].

The evidenced role of these microproteins in physiology, as well as their strong association to different diseases is certainly based on pleiotropic functions that they orchestrate at the cellular level, such as ion channel modulation, DNA repair, RNA expression regulation, cellular senescence, myocyte fusion, mitochondrial metabolism, and others. In contrast, their role in neuron development and neurodegeneration is just emerging, and their therapeutic potential in the brain has not yet been fully addressed [6]. The focus of this review will be to highlight the role of microproteins, with emphasis on mitochondrial microproteins, and their involvement in neurodevelopmental and neurodegenerative diseases. We will also consider the exciting proposition of their potential therapeutic value in the brain.

## 2 Origins and Biological Roles of Microproteins

Microproteins are commonly defined as small proteins consisting of less than 100 amino acids [7]. Unlike classical bioactive peptides, microproteins are not enzymatically cleaved from larger precursors. What's more, microproteins lack a typical N‐terminal signaling region directing them to the secretory route [8], although many microproteins have extracellular functions [9]  and can be secreted from the cell through the secretory pathway (such as demonstrated for the microprotein Humanin) [10]  or associated to circulating extracellular vesicles [11]. They rather reside in the cytoplasm, cellular organelles like mitochondria, and a considerable number of these microproteins localize to the plasma membrane through their hydrophobic domain and to membranes of intracellular organelles, where they regulate many biological processes [12].

**FIGURE 1:**
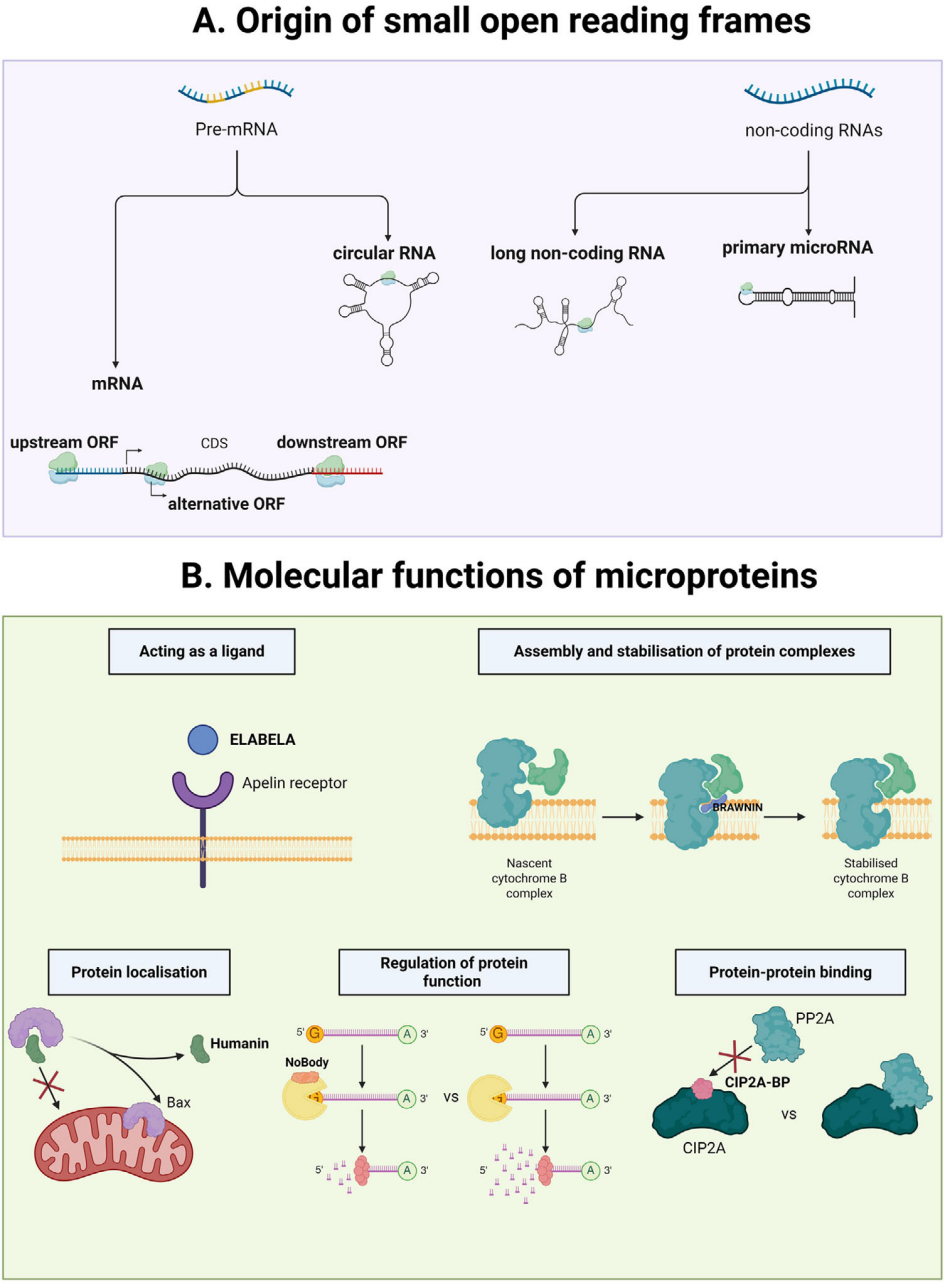
Origin and function of microproteins. (A) Schematic representation of hidden sORF sites inside coding and non‐coding RNAs. Microproteins can be derived from sORFs present inside messenger RNA either in the 5′ or 3′ UTR (upstream or downstream sORFs) or from alternative translation start sites inside the already known coding region. Circular RNA generated by back‐splicing of mRNAs possess sORF and can produce functional microproteins. Previously reported as non‐coding RNAs: long non‐coding RNAs and primary microRNAs (precursors of microRNAs) possess sORF sites in their sequence and can encode microproteins. (B) The functions attributed to microproteins can be grouped in five categories: (i) ligands involved in cell signaling pathways, (ii) scaffolds or assembly factors that allow the construction of large protein complexes, (iii) factors controlling the localization of proteins by binding them, (iv) regulators of enzymes that facilitate or slow down the enzymatic reaction, and (v) inhibitors of protein interaction through competition to binding sites. Created in BioRender (2025) https://BioRender.com.

Microproteins are either derived from (i) sORF in the genome considered as non‐canonical CDS or from (ii) sORF residing within non‐coding RNAs (Figure 1A). Both nuclear and mitochondrial genomes can produce microproteins, even if only 11 mitochondrial produced microproteins have been identified so far [9, 13] (see Table 1). Still, this number shows an enrichment in microprotein‐coding ORFs in the mitochondrial genome owing to its smaller size compared to the nuclear genome (11 verified microproteins for 16,569 bp mitochondrial DNA, compared to 7264 non‐canonical ORFs in 3.1 × 10^9^ bp of nuclear DNA) [9, 14, 15].

**TABLE 1:** Length, origin, localization, biological role and putative clinical roles of MDPs (mitochondrial derived peptides).

Microprotein	Origin	Localization	Biological roles (intracellular)^a^	Putative clinical roles	Length (a.a)	References
***Humanin***	Mitochondrial genome at the 16S ribosomal RNA locus (MTRNR2 gene)	Mitochondria/cytoplasm/secreted	Inhibition of apoptosis by binding to pro‐apoptotic proteins such as IGFBP3 and Bax	Neuroprotective role in Alzheimer disease and in Parkinson disease	21/24	https://doi.org/10.3390/biology12121534
***SHLP1***	Mitochondrial genome at the 16S ribosomal RNA locus (MTRNR2 gene)	N.A.	N.A.	N.A.	24	https://doi.org/10.18632/aging.100943
***SHLP2***	Mitochondrial genome at the 16S ribosomal RNA locus (MTRNR2 gene)	Mitochondria Complex I/secreted	Cytoprotective/enhances mitochondrial metabolism	Protects against amyloid β‐induced toxicity and age‐related macular degeneration, K4R SHLP2 variant protects against Parkinson's disease	26	https://doi.org/10.18632/aging.100943
***SHLP3***	Mitochondrial genome at the 16S ribosomal RNA locus (MTRNR2 gene)	N.A.	Cytoprotective/enhances mitochondrial metabolism	N.A.	38	https://doi.org/10.18632/aging.100943
***SHLP4***	Mitochondrial genome at the 16S ribosomal RNA locus (MTRNR2 gene)	N.A.	N.A.	N.A.	26	https://doi.org/10.18632/aging.100943
***SHLP5***	Mitochondrial genome at the 16S ribosomal RNA locus (MTRNR2 gene)	N.A.	N.A.	N.A.	24	https://doi.org/10.18632/aging.100943
***SHLP6***	Mitochondrial genome at the 16S ribosomal RNA locus (MTRNR2 gene)	N.A.	Apoptotic	N.A.	20	https://doi.org/10.18632/aging.100943
***SHMOOSE***	Mitochondrial genome at the tRNA‐Ser overlapping with a small portion of *nad5*.	IMM	Increases mitochondrial metabolism and interacts with mitofilin	D47N variant associates with Alzheimer's disease SHMOOSE protects from amyloid beta pathology	58	https://doi.org/10.1038/s41380‐022‐01769‐3, https://doi.org/10.1186/s12915‐023‐01609‐y
***MOTS‐c***	Mitochondrial genome at the 12S rRNA region	Mitochondria/nucleus/secreted	Direct regulation of nuclear gene expression, AMPK/NRG1‐ErbB4/Akt pathways activation	Protective effect against cardiac dysfunction, variant K14Q associates with diabetes	16	https://doi.org/10.3389/fendo.2023.1120533, https://doi.org/10.18632/aging.202529
***Gau***	Mitochondrial genome (MT‐CO1 gene)	Mitochondria	N.A.	N.A.	100	https://doi.org/10.1186/1745‐6150‐6‐56
***mtALTND4***	Mitochondrial genome (MT‐ND4 gene)	Mitochondria/cytoplasm/secreted	Decreases mitochondrial respiration and increases ROS production	N.A.	99	https://doi.org/10.1186/s12915‐023‐01609‐y

Abbreviations: IMM, inner mitochondrial membrane; N.A., not applicable.

^a^ This table focuses on the intracellular roles of mitochondrial derived microproteins but several of them are secreted and exert extracellular roles including Humanin or SHLP2.

Microproteins can be translated from the 5′ and 3′ untranslated regions (UTRs) of a transcript including an upstream (uORF) [16] or a downstream open reading frame (dORF), respectively. Of note, many of these sORFs regulate the translation of the main ORFs from which they are produced, that is, the translation of dORFs enhance the translation of their canonical ORFs independently of the dORF‐encoded peptide [17]. Microprotein‐coding genes were also discovered in intergenic regions [18]. In addition, microproteins are abundantly produced from an array of previously considered noncoding RNAs: long noncoding RNAs (lncRNAs) [19], circular RNAs [20], rRNA [21], and pri‐miRNAs [22].

The characterization of microproteins based on size leaves open the question of their functions and which cellular pathways they might more specifically control. Since their identification, many studies have used different approaches to gain insight into the biological functions of microproteins. Targeted studies uncovered a whole palette of biological roles within the cell, including RNA processing, cell death, signaling, and cell communication, among others. Microproteins ensure these fundamental cellular functions through shared molecular functions, modulating protein‐protein interactions, protein localization, or conformation (Figure 1B). As an example, the human microprotein NoBody (non‐annotated P‐body dissociating polypeptide) interacts with mRNA decapping proteins, which remove the 5′ cap from mRNAs to promote 5′‐to‐3′ decay, and regulates their association to the cytoplasmic P‐bodies through a yet unresolved molecular mechanism [23]. The 24 amino acids microprotein humanin (HN) interacts with Bax and prevents its translocation from cytosol to mitochondria providing an anti‐apoptotic protective cellular role [24]. Another microprotein called CIP2A‐BP competes with the protein phosphatase 2 (PP2A) for binding to CIP2A, which facilitates the inhibition of the oncogenic PI3K/AKT/NFκB signaling pathway. CIP2A‐BP also inhibits cancer cell invasion in vivo and correlates with better patient survival [25]. Finally, the zebrafish hormone microprotein Toddler [26] and its human equivalent ELABELA [27] are essential for early cardiac development, both functioning as ligands of the Apelin receptor, a key signaling pathway of the cardiovascular system. A more comprehensive view could be reached by unbiased CRISPR‐mediated screens [28]. Interestingly, these approaches demonstrate that many microproteins are coregulated, and sometimes share function, with the main ORF protein. More recently, databases are being developed, such as MicroProteinDB, to try and provide knowledge on microprotein functions based on structural prediction [29].

As mentioned earlier, functional characterization of microproteins is far from being cataloged. The field still needs extensive investigation analysis to cover the role of the identified microproteins and to validate and reveal the potential biological functions of the broad repertoire of putative microproteins.

## 3 Microproteins in Physiology and Pathology

Despite their recent identification, microproteins rapidly emerged as relevant for human health. Those investigations have led to the discovery of many microproteins playing key roles in physiological processes and in various diseases, although the pathogenic nature of variants in sORFs remains hard to demonstrate on a larger scale [30]. Strategies such as differential proteomics in specific cell lines [31] succeeded to identify new microproteins and to link specific ones to biological conditions. Global microprotein quantitation revealed 12 unannotated and differentially expressed microproteins in two human leukemia cell lines, K562 and MOLT4 [31]. What's more, proteomic analyses at specific developmental time points [32] uncovered 410 microproteins whose expression is differentially regulated during the life cycle of the *Drosophila*. Over half of these microproteins are conserved across species, indicating their critical functions in development [32].

Many microproteins have been related to cancer. Their identification, functional role, and potential clinical application, as well as the mechanisms by which their dysregulation is linked to cancer formation, have been reviewed recently [33, 34]. This can be a consequence of their implication in various cancer‐related processes such as tumor suppression, cell proliferation, and metastasis. As an example, 79‐amino acids small peptide, FORCP (FOXA1‐Regulated Conserved Small Protein), was found to inhibit tumorigenesis in colorectal cancer [35]. Another interesting example is SMIM30 that controls cell migration and proliferation in hepatocellular carcinoma (HCC) and can be targeted in HCC diagnosis, prognosis, and therapy [36]. Interestingly, the above‐mentioned mRNA decapping microprotein NoBody is translated from a lncRNA called LINC01420 that is a potential prognostic biomarker in nasopharyngeal carcinoma, since it correlates with metastasis and a poor prognosis in patients [37]. Thus, all these small molecules open up new therapeutic avenues in fighting cancer progression.

Now, among key organismal processes particularly rich in microproteins appear muscle function and mitochondrial processes [38]. Microproteins can either induce or inhibit muscle contraction, mostly by regulating the Ca2+ flux into the sarcoendoplasmic reticulum (SR) through the SR calcium transport ATPase (SERCA) pump. Myoregulin microprotein is strongly expressed in skeletal muscle and provides a suppressor regulatory effect on SERCA by direct interaction [39]. Yet another microprotein named DWORF promotes SERCA activation by inhibiting different regulatory proteins [40]. Other microproteins have a significant role in myogenesis; for example, Myomixer (also known as Minion) induces the fusiogenic process of myotubes from progenitor cells [41] and is required for satellite cells fusion [42], consequently regulating muscle development and regeneration. A mitochondrial microprotein, Mitoregulin, also called LEMP, is upregulated during muscle differentiation and promotes muscle formation and regeneration [43]. Another well‐conserved microprotein, SPAR, prevents mTORC1 activation and thus inhibits muscle regeneration [44]. Interestingly, the role of these microproteins in the muscle is not just limited to muscle contraction and development, some microproteins have been shown to regulate mitochondrial metabolism in muscle tissue. Mitoregulin controls energy homeostasis through mitochondrial metabolism and thus influences muscle contraction and regeneration [45, 46]. All these findings show the important roles of these microproteins at the crossroads of different pathways regulating muscle physiology and functionality.

Microproteins have also been linked to neurodegenerative diseases. The highly conserved 25 AA ribosomal subunit RPL41microprotein has recently been suggested to be a useful biomarker for Alzheimer's disease [47]. Furthermore, a novel mitochondrial‐encoded microprotein called SHMOOSE has been identified with a mitochondrial variant associated to Alzheimer's disease [48]. Microproteins can regulate synapses, neuroinflammation, neuronal development, and differentiation, showing the relevance of these small proteins as treatment targets in many neuronal diseases. Researchers should also take into consideration the previous works on long non‐coding RNAs and the nervous system as these studies may reveal new neuronal‐related microproteins derived from sORFs on these lncRNAs.

## 4 Mitochondrial Microproteins

Mitochondria are double‐membrane organelles orchestrating several crucial functions in the cell. In addition to being the principal organelle for ATP production through oxidative phosphorylation (OXPHOS), they also have roles in calcium homeostasis, oxidative stress, and apoptosis. Mitochondrial functions rely on massive protein complexes or supercomplexes containing a varying number of proteins and microproteins. Examples of such complexes include TOMM and TIMM complexes for protein importation or MITRAC complex that drives Complex IV assembly in OXPHOS (Makarevich et al., 2023, review).

In recent years, at least two studies have pointed out the relevance of microproteins to the mitochondrion. The first report by van Heesch and collaborators [15] showed a significant enrichment of microprotein‐producing lncRNAs in a cluster of mitochondrial genes in the nuclear genome. The authors were able to tag‐fuse many of these microproteins to validate their mitochondrial localization. In the second report, Zhang et al. showed that the mitochondrion proteome is enriched with microproteins and then identified 173 candidates using a mitochondrial‐localization prediction pipeline. With the same strategy of HA‐tag fusion as in the first report, they succeeded to show that 20 microproteins were clearly displayed in the mitochondria [49].

Although the reason behind mitochondrial enrichment in microproteins remains elusive, two explanations have been put forth: (i) because of their small size, these microproteins are suitable to fit into tight cavities inside the supercomplexes of the respiratory chain [7], or (ii) because of the reduced energy cost for their transport to newly produced mitochondria in low energy conditions [49]. Besides the high number of microproteins that reside in mitochondria, researchers carry a great interest in metabolic aspects of diseases, making a rapid development in the identification of numerous disease‐related mitochondrial microproteins.

Even though interest in mitochondrial microproteins has been booming lately, one should also consider that many small and well‐characterized proteins were already known to be part of the mitochondrial proteome before labeling small proteins as microproteins [50]. MT‐ATP8 is a 68a.a. subunit of the Complex V ATPase that was first described in 1983 [51]. Another example is NOXA protein whose transcription and translation regulation responds to various cellular stresses, and that has been first identified in 1990 [52]. Nowadays, researchers are using the abbreviation MLP for mitochondrion‐located peptides [53], and we will use this same term in our review to pinpoint all, preferentially or exclusively, mitochondrion‐located microproteins whether produced by the nuclear genome or by the mitochondrial genome. MDP (mitochondria derived peptides) refers exclusively to microproteins that are derived from the mtDNA. A list of the main MDPs identified so far can be found in Table 1, and the most recently described MLPs derived from the nuclear DNA are listed in Table 2.

**TABLE 2:** Length, origin, localization, biological role and putative clinical roles of selected MLPs (mitochondria localized microproteins) non derived from mitochondrial DNA.

Microprotein	Origin	Localization	Biological roles (intracellular)	Putative clinical roles	Length (a.a)	References
***Mitoregulin***	lncRNA (LINC00116)	IMM	Extensive: Diverse roles on OXPHOS, fatty acid metabolism, muscle and kidney physiology	N.A.	56	http://doi.org/10.3389/fcell.2025.1545359
***STMP1/Mm47***	lincRNA (1810058I24Rik)	OMM/IMS/IMM	Mitochondrial fission by activating DRP1, enhances mitochondrial Complex IV activity	Controls innate immunity	47	http://doi.org/10.4049/jimmunol.1900791, https://doi.org/10.1016/j.ymthe.2022.04.012
***MOCCI***	C15ORF48 gene	IMM	Replaces NDUFA4 in Complex IV during inflammation and reduces ROS production	N.A.	83	https://doi.org/10.1038/s41467‐021‐22397‐5
***ASAP***	lncRNA (LINC00467)	IMM	ATP synthase assembly and activity	Predicts poor prognosis of colorectal cancer patients.	94	https://doi.org/10.1172/JCI152911
***MP31***	uORF of PTEN	IMM or matrix	Metabolic regulator that controls glycol metabolism by inhibiting mLDH and limits lactate to pyruvate conversion	MP31 is deleted/mutated in 45% of glioblastomas patients	31	https://doi.org/10.1016/j.cmet.2020.12.008
***PIGBOS***	PIGBOS1 gene	OMM	Interacts with ER to regulate the Unfolded Protein Response stress pathway	N.A.	54	http://doi.org/10.1038/s41467‐019‐12816‐z
***MIEF1‐MP***	uORF of MID51 gene	Mitochondrial matrix	Promotes mitochondrial fission, regulates mitochondrial translation	N.A.	70	http://doi.org/10.1021/acs.biochem.8b00726
***BRAWNIN***	C12ORF73	IMM	Is a Complex III assembly factor that regulates its biogenesis and coordinates responses to metabolic stresses, involved in the development of cortical axons	Reduced level of BRAWNIN in post‐mortem brains of autism spectrum disorder patients	71	http://doi.org/10.1016/j.celrep.2022.111204, http://doi.org/10.1038/s41467‐024‐46146‐6, http://doi.org/10.1038/nature20612
***SMIM4***	C3ORF78	IMM	Is a Complex III assembly factor that regulates its biogenesis and coordinates responses to metabolic stresses	One of the 7‐gene signature for lung adenocarcinoma patients prognosis	70	http://doi.org/10.1016/j.celrep.2022.111204, https://doi.org/10.1002/jcla.24190
***TIM8A***	*TIMM8A* gene	IMS	Regulates importation of many proteins and their insertion to IMM, plays an important role in Complex IV assembly	*TIMM8A* variant causes deafness‐dystonia‐optic neuronopathy (DDON) syndrome	97	https://doi.org/10.15252/embr.202256430, https://doi.org/10.1002/mgg3.1121

Abbreviations: IMM, inner mitochondrial membrane; IMS, intermembrane space; mLDH, mitochondrial lactate dehydrogenase; N.A., not applicable; OMM, outer mitochondrial membrane.

The functional analysis of mitochondrial microproteins is still under investigation, yet an important number of these MLPs have been characterized as vital regulators of almost all mitochondrial activities [50]: ETC and ATP production, stress response, signaling, and apoptosis. In addition, many microproteins are also involved in protein importation processes into mitochondria mostly as chaperones of the TOMM (translocase of the outer mitochondrial membrane) and TIMM (translocase of the inner mitochondrial membrane) protein importation complexes [54, 55]. Importantly, a new variant of the TIMM8A chaperone known as small TIMM family was reported to cause deafness‐dystonia‐optic neuronopathy syndrome (DDON) [56], highlighting the importance of these MLPs during neurodevelopment. Interestingly, Kang et al. have shown that hTim8a has evolved a different role in neuronal‐like cells as a Complex IV assembly factor and that its deficiency leads to oxidative stress and a higher apoptotic cell death sensitivity [57].

## 5 Mitochondrial Microproteins in Neurodegenerative Diseases

Despite the recent discovery of a large number of previously unknown microproteins, their involvement in brain functions remains unclear. The fact that sORFs can be derived from long non‐coding RNAs (lncRNA), an RNA subset that is highly prevalent in the brain [58, 59], suggests that microproteins could also be more present in the brain compared to other organs. Given the significant reliance of the nervous system on mitochondrial function (Box 1), it is tempting to speculate that the neural cells are particularly dependent on mitochondrial microproteins to adjust and maintain their physiological function not only at the whole‐cell level, but at the whole‐network level. Indeed, the small size of microproteins makes them easy targets to transcribe and translate, a feat that is particularly useful in an environment that requires a fast adaptation to stimuli [60, 61].

**BOX 1: Metabolic requirement for CNS function and maintenance**

The central nervous system (CNS) represents one of the biggest energetic burdens in the human body. Synaptic activity across the numerous circuits in the brain consumes large amounts of ATP in order to sustain proper neuronal function. The generation of action potential, membrane repolarization, intracellular trafficking, synaptic vesicle recycling, etc. are among the numerous pathways necessary for signal transmission [106]. All of the mechanisms mentioned above rely on a constant supply of ATP, rendering the brain especially sensitive to metabolic insults.

Converging evidence now demonstrate that metabolic regulation is of paramount importance for building functional neural networks during the development of the CNS. In particular, mitochondrial metabolism is highly upregulated during this process. Both transcriptional and translational adjustments allow increased mitochondrial biogenesis and oxidative phosphorylation, which are necessary for neuronal growth and synaptic function. Therefore, a disruption in mitochondrial function in neurons can be deleterious as it undermines the continuous neuronal energy supply.

Interestingly, brain metabolism goes through a metabolic decline as it ages. A decrease in brain glucose uptake is one of the earliest signs of Alzheimer's disease (AD) [107, 108] and Huntington's disease (HD) [109, 110, 111], years before the first clinical symptoms manifest. Moreover, almost all neurodegenerative diseases have been linked with mitochondrial deficiency such as reduced mitochondrial respiration, increased oxidative stress, and dysregulations of calcium homeostasis. As a matter of fact, metabolic deregulation represents one of the key hallmarks of neurodegenerative diseases [112]. These findings highlight the importance of preserving mitochondrial activity to protect the brain from the effects of aging.

Until recently, most of the knowledge concerning the involvement of mitochondrial microproteins in the brain was restricted to the field of aging and neurodegeneration. Several groups discovered previously unknown MDPs including Humanin, MOTS‐c, SHLPs 1–6 (Small Humanin‐Like Peptides), and SHMOOSE. Below, we will focus on those that localize to mitochondria and affect mitochondrial function.

There are six microproteins belonging to the group of SHLPs [62]. Their roles are still poorly described especially in the context of the nervous system. SHLP2 is the only microprotein of the group whose role has been characterized in the brain. SHLP2 can be found inside Complex I of the mitochondrial respiratory chain or be secreted in the bloodstream [63]. It has been shown that SHLP2 regulates whole‐body metabolism by reducing food intake and enhancing energy expenditure via the activation of pro‐opiomelanocortin (POMC) neurons inside the hypothalamus [64]. The long non‐coding RNA ASncmtRNA‐1/2 that encodes SHLP2 contains a mitochondrial SNP (mtSNP) m.2158 T > C, which encodes a K4R variant of SHLP2 protein. K4R protein has a longer half‐life and is protective against mitochondrial stress in vitro. A meta‐analysis of mitochondrial DNA has associated this mtSNP with a reduced risk of developing Parkinson disease (PD). In mice exposed to mitochondrial Complex I inhibitor (MPTP) to model PD, the loss of tyrosine hydroxylase and dopamine was partially negated with K4R SHLP2 pretreatment [63]. In addition, SHLP2 treatment prevents Aβ cytotoxicity in ARPE‐19 cybrid cells containing mitochondria derived from age‐related macular degeneration (AMD) patients [65].

The microprotein SHMOOSE localizes to mitochondria and interacts with mitofilin (a regulator of cristae structure and inner mitochondrial membrane organization). SH‐SY5Y cells treated with exogenous SHMOOSE demonstrate reduced levels of mitochondrial superoxide and increased maximal mitochondrial respiration capacity [48]. These responses are dependent on the interaction between SHMOOSE and mitofilin. Downregulation of mitofilin in SH‐SY5Y cells exposed to extracellular SHMOOSE abolishes the aforementioned effect. Intriguingly, SHMOOSE RNA levels are increased in the brain of AD patients despite the global decrease of metabolic activity in the AD brain. The upregulation of SHMOOSE could be a compensatory mechanism to limit the cellular damage. Additional data suggest that SHMOOSE can be protective against Aβ cytotoxicity in vitro. Interestingly, a pathogenic variant of SHMOOSE D47N is suggested to increase the risks of AD development 20%–50% making it a potential biomarker of the disease [48]. This is likely due to altered mitochondrial SHMOOSE function, which has not been fully demonstrated yet.

Finally, it is worth noting that MOTS‐c, although it does not directly localize to the mitochondria, affects cellular metabolism by interfering with the AMPK pathway [66]. MOTS‐c decreases with age, and its re‐expression can improve memory deficits in a mouse model of Alzheimer's disease [67]. Currently, synthetic analogs of MOTS‐c are ongoing clinical trials for metabolic diseases [68], although their potential in neurodegenerative diseases has not yet been explored.

## 6 Mitochondrial Microproteins in Neurodevelopment

**FIGURE 2:**
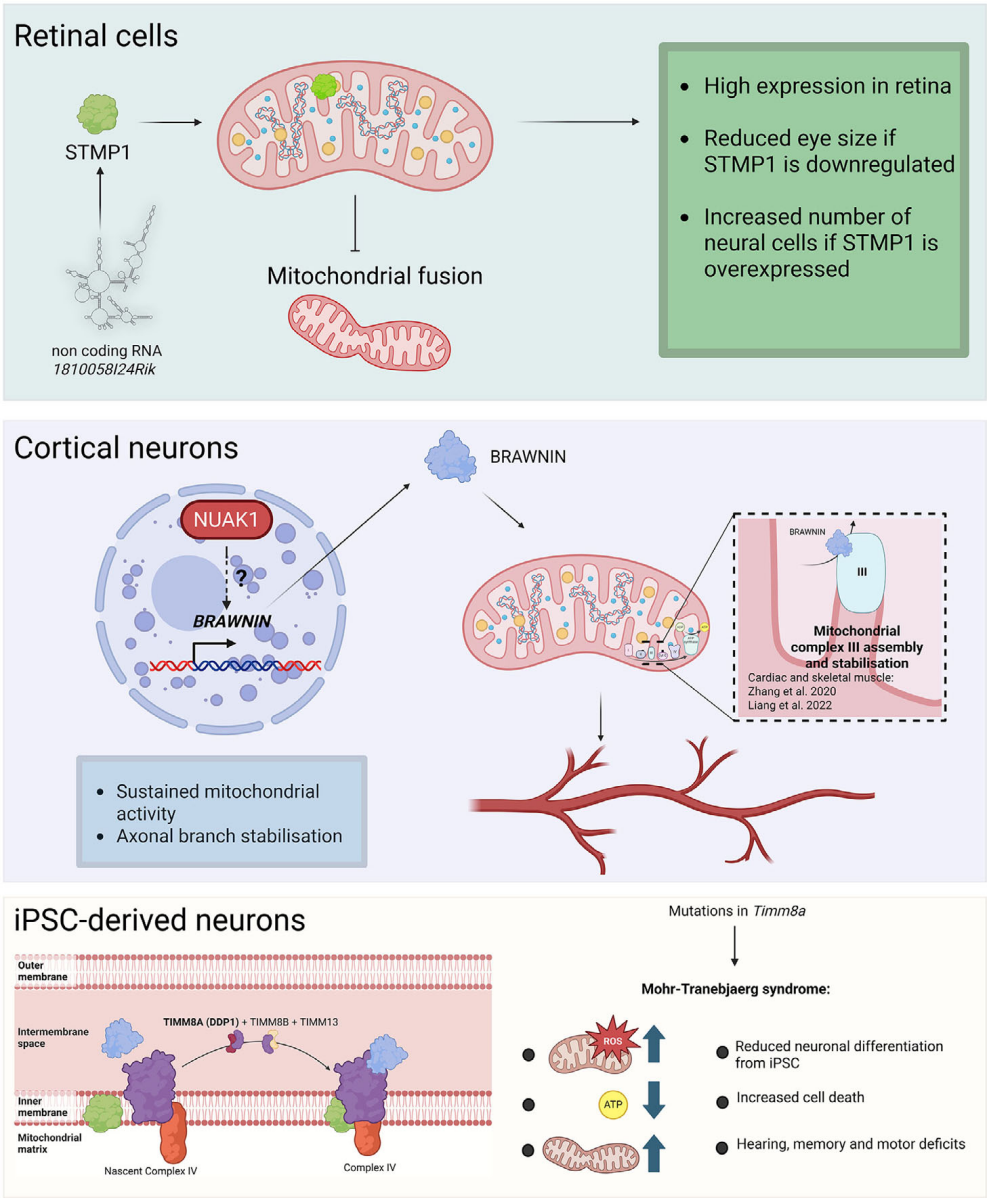
Mitochondrial microproteins in neurodevelopment. A schematic representation of the roles of STMP1 (above), BRAWNIN (middle), and TIMM8A (below) in neural development. Above: STMP1 is encoded by a long non‐coding RNA *1810058124Rik* and is inserted inside the inner mitochondrial membrane and negatively regulates mitochondrial fusion. The upregulation of STMP1 in the mouse retina increases the proportion of different neuronal and glial cell types. The downregulation of STMP1 reduces the eye size in Zebrafish. Middle: The coding sequence of *Brawnin* is hidden inside the *Tdg* gene. *Brawnin* is regulated by a Serine/Threonine kinase NUAK1. Upon translation, it is translocated to the inner mitochondrial membrane to regulate the assembly of the III complex of the mitochondrial respiratory chain to sustain normal mitochondrial activity necessary for axonal branch development. Below: TIM8A is located inside the intermembrane space inside of mitochondria. Along with TIM8B and TIM13, it allows the assembly of the IV complex of the respiratory chain. Mutations in *TIMM8a* in humans result in the Mohr‐Tranebjaerg or deafness‐dystonia‐optic neuropathy syndrome (DDON), a neurological disorder. Neurons derived from human iPSCs demonstrate a mitochondrial defect as well as alterations in neuronal differentiation. In particular, *TIMM8A* mutant neurons are smaller in size and fail to form a complex neuronal network and present a higher risk of cell death. Created in BioRender (2025). https://BioRender.com/.

As opposed to neurodegeneration, the roles of mitochondrial microproteins in the field of neurodevelopment have been overlooked until fairly recently. One example of a mitochondrial microprotein with importance in CNS development is STMP1 (Short transmembrane Mitochondrial Protein 1), a transmembrane microprotein that negatively regulates mitochondrial fusion and activates the Nlrp3 inflammasome pathway [69]. STMP1 RNA levels are enriched in the brain of adult zebrafish. Loss of STMP1 is beneficial in ischemia‐reperfusion injuries and limits the microglial response to avoid excessive tissue damage. It is highly expressed across all retinal tissue during development in the mouse embryo. Overexpression analysis shows that STMP1 increases the proportion of amacrine (interneurons), bipolar cells (excitatory neurons), and Müller cells (glia) [70]. Knockdown of STMP1 in zebrafish reduces the eye size and respiratory ventilation frequency [71]. Although further investigation is necessary, STMP1 could play a role in the regulation of neuronal differentiation inside the retina based on the aforementioned observations (Figure 2).

BRAWNIN (UQCC6/C12ORF73/Sloth2) is an evolutionary‐conserved mitochondrial microprotein that is necessary for the assembly and stabilization of the Complex III of the ETC [49, 72]. BRAWNIN is located inside the IMM, where it forms the COMB complex in collaboration with SMIM4 and C16ORF91 proteins. COMB is necessary for the insertion of cytochrome B inside the IMM (a crucial step for the biogenesis of the Complex III). Loss of BRAWNIN results in Complex III destabilization and reduces the OxPhos function of mitochondria leading to reduced mitochondrial potential and respiration [49, 73]. In animal models, the loss of BRAWNIN results in major defects at the level of the whole organism such as growth retardation, neurodegeneration, and premature death in zebrafish [49] and Drosophila [73]. Although the brain phenotype was not studied in mice deficient for BRAWNIN, these animals present low exercise tolerance showing the importance of BRAWNIN in a whole‐body physiological response [72].

To maintain the bioenergetic status of the cell, BRAWNIN is quickly upregulated in response to metabolic stress (such as nutrient starvation) [49]. At the transcriptional level, BRAWNIN is regulated by NUAK1, a serine/threonine kinase related to the metabolic regulator AMPK [74] and whose function is needed to support cortical axon development [75, 76]. We recently demonstrated that NUAK1 supports axonal mitochondrial function through the regulation of *Brawnin* gene expression and mRNA alternative splicing [77]. Consequently, downregulated mitochondrial function in *Nuak1* KO and in *Brawnin* KD leads to a reduced number of axonal branches both in vitro and in vivo (a process highly dependent on mitochondria). Remarkably, increasing the levels of BRAWNIN restores axonal morphology and mitochondrial activity in *Nuak1* KO neurons. Thus, BRAWNIN is necessary and sufficient to support axonal mitochondrial function, and plays a major role in the stabilization of axonal collateral branches during the formation of cortical circuits [77].

These morphological alterationslikely result in synaptic dysfunction decreasing the excitatory signal of neurons seen in Drosophila *Brawnin* KD animals [73]. The aforementioned defects at the cellular scale could be at the origin of the animal lethality, reduced locomotion in Drosophila [73], and some of the altered behavioral phenotype in NUAK1 haploinsufficient mice [76], although this remains to be studied. The NUAK1 haploinsufficient mice present reduced interest towards social novelty, abnormal sensorimotor gating, and memory consolidation. Large‐scale analysis of gene expression in the post‐mortem brains of idiopathic autism spectrum disorder (ASD) individuals showed reduced levels of BRAWNIN [78]. These findings suggest that it is a crucial player in the development and maintenance of the nervous system and could be implicated in the pathogenic processes leading to neurodevelopmental disorders. Although the clinical implications of BRAWNIN remain unexplored to date, the fact that BRAWNIN expression positively regulates cortical connectivity in a mouse model of altered axonal development [77] suggest it could have a therapeutic potential in neurodevelopmental and neuromuscular diseases.

TIM8A/DDP1 (Deafness Dystonia Peptide), encoded by the *TIMM8A* gene, is an intermembrane space mitochondrial microprotein associated to a rare disease called Mohr‐Tranebjaerg syndrome (MTS), also known as deafness‐dystonia‐optic neuronopathy (DDON) [79]. TIM8A expression is enriched in the liver and in the brain, particularly during embryonic development. Together with TIM13, TIM8A forms a heterohexamer, which plays a crucial role in mitochondrial Complex IV assembly [80]. TIM8A and TIM13 allow the transition from Supercomplex 2 (S2) to Supercomplex 3 (S3) by stabilizing the insertion of COX2 multiprotein structure inside the S2 [81]. The loss of TIM8A results in an incomplete assembly of Complex IV, accompanied with more fragmented mitochondria, increased ROS production, and higher sensitivity to cell death [57, 81, 82]. Remarkably, it seems that the function of TIM8 proteins is not evolutionary‐conserved. It has been shown that its function in yeast revolves around the import of TIM23 inside the intermembrane space [83]. This role is not found in human cells, suggesting that TIM8 function evolved across species [57].

MTS, which results from mutations in *TIMM8A*, is a progressive neurological disorder that manifests itself in early childhood. It is characterized by hearing impairment, dystonia, vision difficulties, and later on dementia. These clinical manifestations have been recapitulated in mouse models, where human‐specific *Timm8a* mutations were introduced in the mouse genome [84]. Recent findings in neurons derived from patient induced pluripotent stem cells (iPSCs) showed alterations in the neurodevelopmental program of TIMM8A mutant cells [82]. In particular, neural progenitor cells (NPC) have decreased levels of NPC markers such as Nestin, PAX6, and SOX2, suggesting a defect in the differentiation process. Furthermore, TIMM8A‐deficient neurons show reduced soma size and reduced number of neurites. In addition, certain phenotypic abnormalities such as increased oxidative stress, cell death sensitivity, and reduced soma area can be partially rescued by either Vitamin E treatment (antioxidant) or CHCHD2 overexpression (a mitochondrial protein involved in response to ROS and apoptosis) in *TIMM8A* mutant cells. Overall, available data demonstrate that TIM8A is an important factor necessary for the proper function of the electron transport chain that is directly linked to a rare disease. The above‐mentioned findings suggest that MTS could be a neurodevelopmental disorder that leads to an early‐onset neurodegeneration.

Although, to our knowledge, BRAWNIN, TIM8A, and STMP1 are the only examples of mitochondrial microproteins linked directly with the development of the CNS, it would not be surprising that many more remain to be discovered, given the important role that mitochondria play in neurodevelopment and the particular enrichment of microproteins at the ETC. As an example, the mitochondrial microprotein MP31 acts as a tumor suppressor in glioblastoma and has been shown as a regulator of the lactate to pyruvate conversion in neural stem cells (NSCs) by inhibiting the activity of the lactate dehydrogenase B [85]. Loss of MP31 in NSCs rewires their metabolism, increasing the production of TCA intermediates, ATP levels, and cell viability while decreasing the mitochondrial membrane potential. Although it has not been demonstrated, it is likely that MP31 influences neural differentiation and maturation since the regulation of both glycolytic and mitochondrial metabolism affects the cell fate of NPC and developing neurons [86, 87].

Currently available literature does not provide more information concerning the involvement of mitochondrial microproteins in the development of the nervous system. However, several groups have demonstrated the importance of microproteins not linked to mitochondria in the establishment of the nervous system. Although the exact role of these proteins is not always determined, they have been linked to neuronal metabolic regulation, chromatin modulation, and calcium dynamics. As an example, the evolutionary‐conserved microprotein pTUNAR (BNLN) is located at the membrane of the endoplasmic reticulum and regulates calcium release and reuptake likely via its interaction with the SERCA2 pump [88]. It is found at the protein level exclusively inside the CNS and is particularly prevalent inside the spinal cord. pTUNAR negatively regulates cortical neurite outgrowth both in vitro and in vivo when overexpressed.

Linc‐mipep (Lnc‐rps25) and linc‐wrb are lncRNA‐derived microproteins highly enriched in early stages of zebrafish developing embryos. Both of these peptides are homologous to High Mobility Group Nucleosome binding protein 1 (HMGN1), which in humans sustains open chromatin structure for transcription. Loss of either of them alters chromatin accessibility in zebrafish larvae brains and results in locomotor hyperactivity likely via reduction of NMDA receptor activity [89].

Although the exact role of SMIM45 has not been discovered, it is known to be involved in human cortical organoid development. Loss of SMIM45 results in premature NPC differentiation and progenitor pool reduction, which ultimately reduces the organoid size [90].

Malat1 is a non‐coding RNA that contains a sORF that encodes a protein. Malat1 is found in the nucleus, but upon neuronal differentiation and maturation, fractions of Malat1 are exported into the cytosol, where it co‐localizes with RNA granules, mostly in the axon. Interestingly, the downregulation of Malat1 protein product affects the global levels and puncta numbers of presynaptic and postsynaptic proteins such as synaptophysin and PSD95 suggesting a potential alteration of synaptic function in these neurons [91]. Future studies will elucidate the exact effect of Malat1 on neuronal development and the molecular mechanisms underlying it.

## 7 Perspective: A Role for Mitochondrial Microproteins in Human Brain Evolution

**FIGURE 3:**
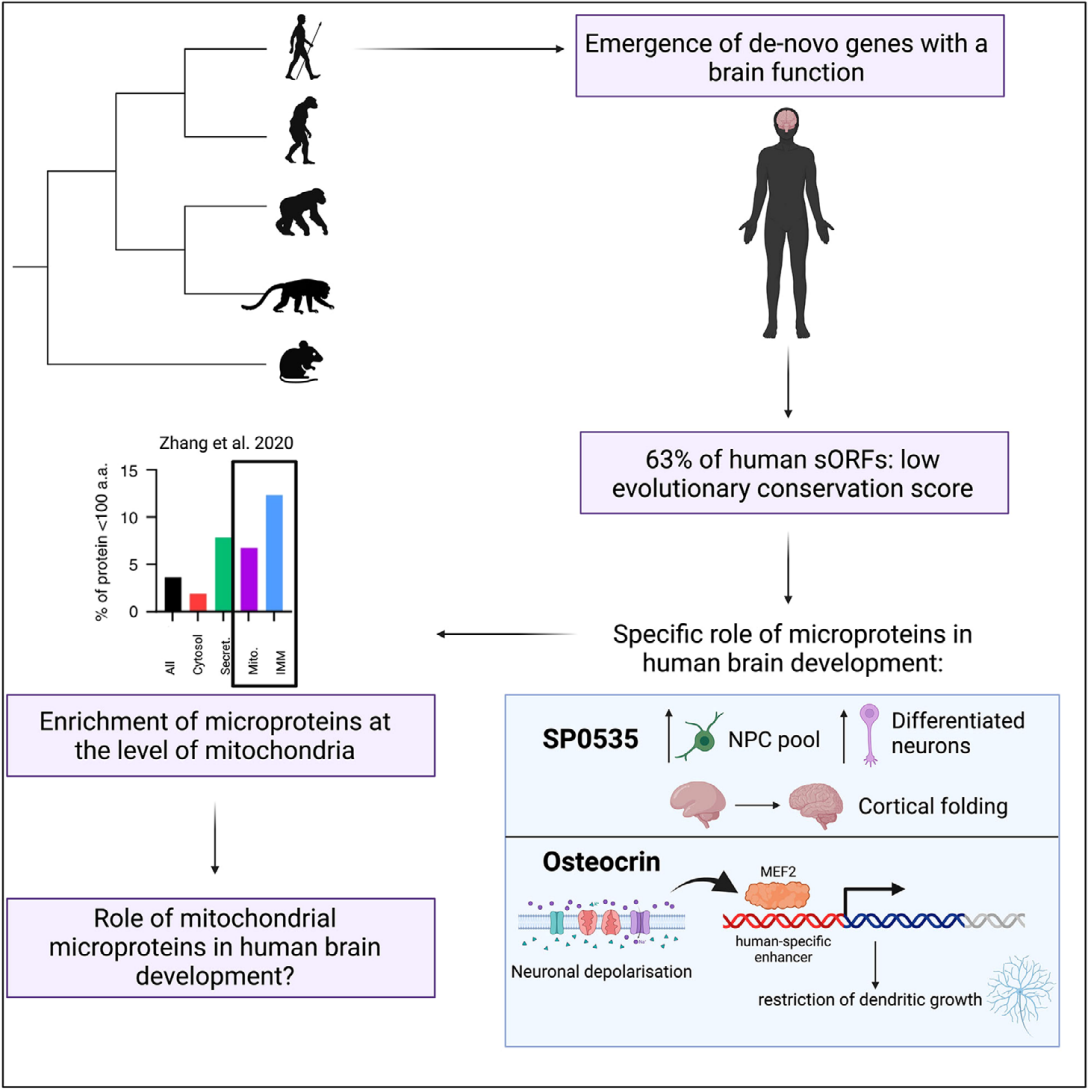
Human‐specific emergence of microproteins. sORFs have a low conservation during evolution and may have contributed to the emergence of species‐specific neurological features. Two examples, SP0535 and Osteocrin, are cited to support the putative role of microproteins in human‐specific brain development. Created in BioRender (2025). https://BioRender.com.

With the advent of evolutionary genomics and the development of modern tools for genome sequence analysis, several examples of human‐specific genes with essential roles in brain development and function have emerged [92]. Importantly, the regulation of mitochondrial dynamics and function play an important role in human‐specific neuron development [93]. Microproteins, and among them mitochondrial microprotein, constitute therefore a vast reservoir of putative human‐specific modifiers of brain development, whose implication remains so far terra incognita (Figure 3).

Computational analysis of recently‐generated databases of translated sORF (ribosome profiling via Ribo‐Seq) suggests that the majority of microproteins could be species‐specific and, thus, have low conservation levels across evolution [94, 95]. Interestingly, less than 20% of human sORFs are conserved in mice, and less than 2% are conserved in older species such as worms and zebrafish [94]. Approximately 63% of human microproteins are suggested to have emerged de novo from non‐coding sequences. An additional study demonstrated that there is a particular enrichment in de novo human genes in general in the brain at the transcriptional level [90]. The emergence of new genes is one of the evolutionary mechanisms underlying the more complex development and structure of the human cerebral cortex. It is reported that most of these genes are functional during the process of cortical development. Among them, there are *SRGAP2C* [96, 97], *ARHGAP11B* [98], or *NOTCH2NL* [99, 100]. In general, the protein products resulting from these genes allow for longer expansion of the neuronal progenitor pool, a higher number of differentiated neurons, and differential synaptic maturation, thus leading to a higher complexity of neural circuits. Studies so far implicated gene duplications and retrotranspositions as the main mechanisms driving this evolution. However, the appearance of new genes from non‐coding regions has been overlooked so far. Recently, a human‐specific microprotein, SP0535, was discovered. It expands the NPC pool and increases the number of differentiated neurons and controls the process of cortical folding. When expressed in mice, SP0535 brings about the formation of gyri‐like structures and increases the performance of mice in spatial and working memory behavioral tasks [101]. An additional example is Osteocrin/Musclin. Although it is a small‐size protein, it does not fit the 100 AA cut‐off criteria of microproteins because of its size of 130AA. However, research has shown that a modification in its enhancer sequence that appeared in primates puts the expression of Osteocrin under MEF2 control [102]. This change renders Osteocrin expression activity‐dependent and is necessary to restrain the dendritic growth in human cortical neurons upon depolarization. This is likely mediated by the interaction between Osteocrin and NPR3 (natriuretic peptide receptor 3) that reduces the intracellular levels of cAMP [103]. Based on these findings, we can speculate that microproteins could be one of the contributors to corticogenesis that allows the primate species to obtain higher cognitive functions.

Further discoveries have shown that global cellular processes such as protein degradation [104, 105] and mitochondrial respiration are functioning at a different rate in human cells when compared to mouse. In particular, human neurons display lower mitochondrial activity that slows down their developmental timing [93]. As mentioned above microproteins are found in large numbers inside mitochondria [49] and play an important role in regulating and fine‐tuning mitochondrial homeostasis. Given the extreme dependence of the nervous system on mitochondria as a constant energy source and a hub of numerous cellular pathways, we can hypothesize that mitochondrial microproteins could be essential for human/primate‐specific brain development.

## 8 Limitations and Future Directions

Despite the technical advances in genome analysis, the unbiased identification of low‐abundance microproteins at the protein level remains a challenge. Insufficient sensitivity, especially of mass‐spectrometry based methods, and lack of standardized pipelines, makes it harder to compare the available studies. Different strategies such as a combination of RiboSeq with MS, are warranted, specifically for mitochondrial proteins to validate experimentally the existence of new microproteins and uncover their roles in physiology and pathology.

Microproteins exhibit a low conservation score across evolution. As a consequence, studies in animal models can be less effective owing to a lack of sequence conservation. Furthermore, human neurons and brain tissues have very limited accessibility; thus, iPSCs induced into neuronal progenitor cells (NPC) and differentiated to different types of neurons (iNs) constitute a relevant model to identify and characterize neuronal‐related microproteins. In the long run, mitochondrial microproteome profiling in diseased iNs will uncover disease‐associated mitochondrial microproteins of therapeutic interests, either as biomarkers or therapeutic targets.

In conclusion, mitochondrial microproteins could have a high therapeutic potential in a wide range of diseases, including conditions affecting neuronal development and neurodegeneration. Especially, the small size of microproteins make them amenable to therapeutic manipulation, such as vectorization to improve stability and biodistribution. Several analog peptides for microproteins are currently ongoing pre‐clinical or clinical trials in a variety of diseases such as obesity, Cancer, or Alzheimer's disease. Given the pleiotropic roles of mitochondria in health and disease, mitochondrial microproteins represent a promising therapeutic avenue in a wide range of diseases of the central nervous system.

## Author Contributions

N.B. and S.Y. drafted the manuscript and figures. J.C. initiated and supervised the work. All authors edited and approved the manuscript and figures.

## Conflicts of Interest

A patent application EP 23 305 322 entitled “BRAWNIN AGONISTS FOR USE IN THE TREATMENT OF AXONAL METABOLIC DISORDERS” was filed on March 9, 2023 (S.Y. and J.C.).

## Data Availability

Data sharing not applicable to this article as no datasets were generated or analyzed during the current study.
